# Neural crest and sons: role of neural crest cells and Schwann cell precursors in development and gland embryogenesis

**DOI:** 10.3389/fcell.2024.1406199

**Published:** 2024-06-26

**Authors:** Aleksandra Knyazeva, Vyacheslav Dyachuk

**Affiliations:** Almazov Federal Medical Research Centre, Saint Petersburg, Russia

**Keywords:** neural crest stem cells, pluripotency, cellular hierarchy, Schwann cells, gland development normal

## Abstract

In this review, we consider the multipotency of neural crest cells (NCCs), Schwann cell precursors (SCPs), and their role in embryogenesis base on genetic tracing and knock out model animals and single cell transcriptomic analysis. In particular, we summarize and analyze data on the contribution of NCCs and SCPs to the gland development and functions.

## Introduction

### Multipotency of neural crest cells and Schwann cell precursors

**FIGURE 1:**
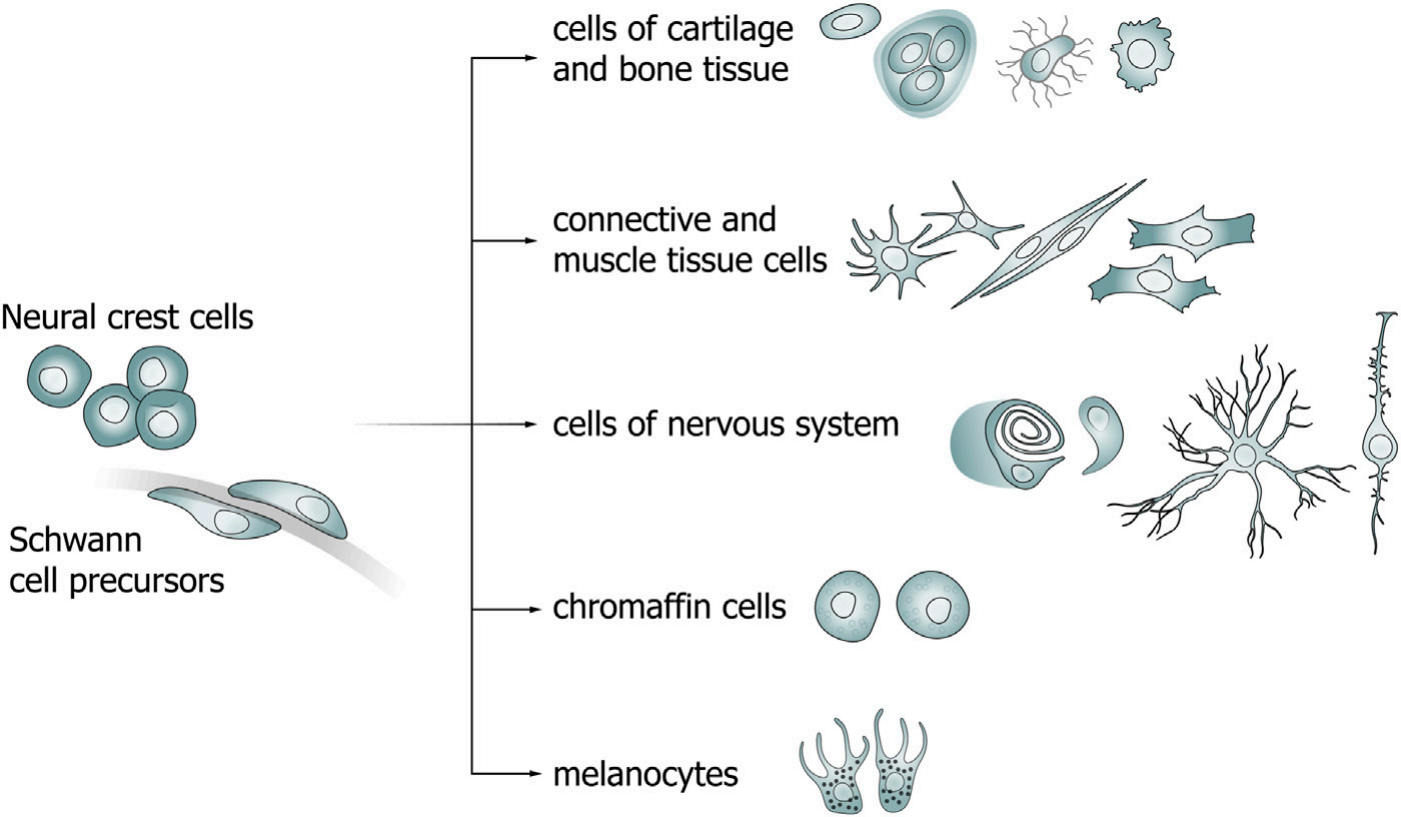
Diagram of neural crest cell (NCC) and Schwann cell precursor (SCP) differentiation pathways.

The neural crest is a population of multipotent migratory cells that are detached from the border of the neural plate during neurulation and differentiate into cells of various organs in the adult organism (Figure 1; Table 1) (His, 1868). According to the New Head hypothesis, it was the appearance of the neural crest and epidermal placodes that led to the diversification and widespread distribution of chordates (Gans and Northcutt, 1983; Martik and Bronner, 2021). Neural crest cells undergo the stage of epithelial–mesenchymal transition and begin migrating to distant areas of the body. The contact of NCCs with growing peripheral nerves, as well as a change in the transcriptional signatures (*Snai1, Sox9/10*, *Foxd3, Pax3* and others for NCCs; *Sox10, Sox2, NRG1* for SCP), results in the formation of Schwann cell precursors, whose developmental direction depends on the diameter of the axon along which they migrate.

**TABLE 1:** Cell types derived from neural crest cells and Schwann cell precursors.

NCCs/SCPs descendants	References
Smooth muscle cells	Minoux et al. (2009), Arima et al. (2012)
Chondrocytes and osteoblasts	Xie et al. (2019)
Melanocytes	Adameyko et al. (2009), Kaucka et al. (2021)
Pulp cells and odontoblasts	Kaukua et al. (2014)
Sympathetic neurons	Kastriti et al. (2019)
Parasympathetic neurons	Dyachuk et al. (2014)
Sensory neurons	Ito and Sieber-Blum (1991)
Enteric neurons	Nishiyama et al. (2012), Hutchins et al. (2018)
Promyelinating Schwann cells	Feltri et al. (2016), Michailov et al. (2004), Taveggia et al. (2005)
Remak cells	Monk et al. (2015), Gomez-Sanchez et al. (2017)
Müller glia	Turner and Cepko (1987), Wetts and Fraser (1988)
Neurons and glia in ovaries	McKey et al. (2019)
Glomus type I cells of carotid bodies	Hockman et al. (2018)
Chromaffin cells of adrenal medulla and organ of Zuckerkandl	Furlan et al. (2017), Kamenev et al. (2021), Kastriti et al. (2019)
Prostate neuroendocrine cells	Szczyrba et al. (2017)
Pericytes and all hormone-producing cell lineages of adenohypophysis	Ueharu et al. (2018), Kato et al. (2021)
Perivascular cells in thymus	Foster et al. (2008)
Connective tissue of different glands	Nakamura (1982), Suinara et al. (2000), Müller et al. (2008), Johansson et al. (2015), Takahashi et al. (2014), Jaskoll et al. (2001)

Depending on the place of their origin and directed migration, the entire population of neural crest cells (NCCs) can be divided into several groups: cranial, trunk, cardiac, and vagal NCCs (Achilleos and Trainor, 2012).

In mammals, cranial NCCs give rise to the cartilage and bone structures of the jaws and inner ear (Couly et al., 1998; Freyer et al., 2011). In addition, this cell population gives rise to the tooth’s dentin, the bones of the frontonasal process, and the peripheral neurons and glia of the cranial nerves (Le Lièvre, 1978; Chai et al., 2000; Méndez-Maldonado et al., 2020). The skeletogenic potential of the cranial neural crest has been extensively studied and documented from vertebrates, although cells of the trunk neural crest may also contribute to skeletal components in some animals like the contribution of NCCs to the differentiation of the plastron bones (abdominal carapace bones) of the turtle *Trachemys scripta* (Cebra-Thomas et al., 2007). The cranial NCCs also differentiate into the Müller glia that play an important role in the architecture and proper functioning of the retina (Turner and Cepko, 1987; Wetts and Fraser, 1988). NCCs in the skull produce a variety of factors that regulate the proper development of brain regions (Le Douarin et al., 2007; Le Douarin et al., 2012). The post-otic cranial NCCs form the aortopulmonary septum and also differentiate into smooth muscle cells of the third, fourth, and sixth arteries of the pharyngeal arches (Minoux et al., 2009; Arima et al., 2012). Ablation of the post-otic part of the cranial neural crest located above somites 1–5 showed that the thymus gland, parathyroid glands, and thyroid glands are underdeveloped or not formed (Bockman and Kirby, 1984). In this regard, the cells of the post-otic part of the cranial neural crest are suggested to play an important role in the morphogenesis not only of the cardiovascular system but also of the endocrine glands (Maeda et al., 2016).

The cardiac NCCs are involved in cardiovascular development in several ways: by remodeling the aortic arch arteries, by dividing the cardiac outflow tract, and by regulating the availability of factors required for normal aortic positioning and functional myocardial maturation in the caudal pharyngeal region (Lee and Saint-Jeannet, 2011). Ablation of the cardiac neural crest resulted in persistent truncus arteriosus (PTA), aplasia or hypoplasia of the thymus, thyroid, and parathyroid glands, and defects in the aortic arch arterial remodeling. Also, the ablation of the cardiac neural crest indirectly led to defects in myocardial function. In studies on quails, a clonal analysis of derivatives showed that cardiac neural crest cells can give rise to melanocytes (Adameyko et al., 2009), smooth muscle, chondrocytes (Xie et al., 2019), connective tissue, and sensory neurons (Ito and Sieber-Blum, 1991).

NCCs migrate to the areas of the forming organs (including the heart) and differentiate into intramural neurons and peripheral glia. Cells from the vagal, trunk, and sacral regions of the neural crest migrate into the intestinal wall, giving rise to enteric neurons and glia from all parts of the intestine. Using frame-by-frame imaging analysis of mouse enteric neural crest cells (ENCCs), Nishiyama et al. (2012) showed that a population of ENCCs moves from the midgut to the hindgut via the mesentery during embryogenesis and that such “transmesenteric” ENCCs make up a major part of the enteric nervous system of the hindgut. This migratory process requires GDNF signaling, and there is evidence suggesting that impaired transmesenteric migration of ENCCs may underlie the pathogenesis of Hirschsprung’s disease (intestinal agangliosis).

The vagal NCCs originating from the neck region colonize the intestine, forming ganglia of the enteric nervous system that control intestinal peristalsis (Hutchins et al., 2018). Chicken trunk NCCs showed the ability to give rise to teeth when implanted into the epithelium of the mouse mandibular arch (Lumsden, 1988). Furthermore, an increase in the level of the transcription factor (TF) microphthalmia (Mitf), which is key TF in melanocyte differentiation, in the trunk NCCs was observed on about day 9.5 of embryonic development, immediately prior to migration. The expression level of the melanogenic enzyme dopachrome tautomerase (Dct) was also found to be elevated in these cells. This enzyme is a marker of melanocytes at all stages, including melanoblasts (MacKenzie et al., 1997; Goding, 2000; Nishimura et al., 2002; Adameyko et al., 2009).

One of the ways by which NCCs differentiate is becoming Schwann cell precursors (SCPs) that also have the ability to differentiate into a wide range of cells (Figure 1) (Furlan and Adameyko, 2018; Kastriti et al., 2022). Primarily, SCPs give rise to myelinating and non-myelinating Schwann cells in the peripheral nervous system (PNS). The transition from NCCs to SCPs is a consequence of the interaction of the former cells with axons (Monk et al., 2015). The molecular mechanisms that direct a portion of the SCPs population to peripheral nerves and the causes of selection of specific nerve types are not fully understood. The transition from SCPs to immature SCs (imSCs) is accompanied by an important mechanistic step involving the selection of axons for myelination (radial sorting) of imSCs which, in turn, differentiate into promyelinating SCs (Feltri et al., 2016). The remaining imSCs are connected to axons of smaller diameters and differentiate into non-myelinating SCs (Remak cells) (Monk et al., 2015; Gomez-Sanchez et al., 2017). The choice of imSCs to differentiate into myelinating or non-myelinating SCs is determined by the expression levels of neuregulin 1 (NRG1). While lower levels of NRG1 released by a relatively smaller axon lead to the maturation of imSCs into non-myelinating SCs, higher levels lead to the development of myelinating SCs (Michailov et al., 2004; Taveggia et al., 2005; Gomez-Sanchez et al., 2017).

In addition, SCPs are capable of transforming into neurons of internal organs (e.g., intramural neurons of the intestine). It is known that SCPs and Schwann cells originating from the cranial region of the neural crest, forming the dental mesenchyme, take part in the development, growth, and regeneration of teeth. Moreover, SCPs, like NCCs, contribute to the formation of many parts of the skeleton (e.g., mandible and ribs), as well as nasal and auricular cartilage (Xie et al., 2019).

Evidence confirming the ability of SCPs to specialize into other cell types has been obtained by demonstrating their differentiation into melanocytes of the skin (Adameyko et al., 2009) and into extracutaneous melanocytes of the heart, inner ear, and brain membranes (Kaucka et al., 2021). Quite a large number of studies confirm the contribution of SCPs to the formation of melanocytes in various body structures. For example, after the induction of recombination between stages E9.5 and E10.5 in adult mice of the Plp1^CreERT2^/R26^YFP^ line, fluorescent signal was detected in the brain membranes, spinal ganglia, spinal cord and brain, and in the supraorbital spaces between the eyeballs. The same team of researchers analyzed internal organs of several fish and amphibian species for the presence of extracutaneous melanocytes and found an association of pigment cells with blood vessels and nerves.

As shown in a study on SCPs *in vitro*, these cells can differentiate into melanocytes if the activity of protein kinase C changes (Hess et al., 1988). There was also a study on NCCs of avian embryos which showed that endothelin 3 *in vitro* promotes the transition of Schwann cells into melanocytes (Dupin et al., 2003). The authors suggested that glial cells and melanocytes share a common precursor. They also stated that glial cells and pigment cells that originated from NCCs are phenotypically unstable *in vitro* and can revert to a bipotential precursor state. Another study on avian NCCs showed that bFGF can promote transdifferentiation of adult Schwann cells into melanocytes (Sherman et al., 1993). It is important to note that the authors used HNK-1 as a marker of Schwann cells with the neural crest origin. Subsequently, it turned out that this molecule cannot be used as a marker of neural crest cells and, therefore, the results obtained require additional verification.

Another example of multipotency of SCPs is their ability to give rise to parasympathetic neurons (Dyachuk et al., 2014). Genetic tracing experiments on transgenic mice, performed by two independent research teams, have convincingly demonstrated that parasympathetic neurons in cranial ganglia, intramural (interstitial) ganglia of the heart, and sacral parasympathetic ganglia after E12.5 originate from nerve-associated SCPs (Dyachuk et al., 2014; Espinosa-Medina et al., 2014). SCPs are capable of differentiating into enteric neurons during postnatal neurogenesis (Uesaka et al., 2015). Tracing using lipophilic dyes and the Sox10Cre inducible system in a study on *D. rerio* has shown that postembryonic enteric neurons arise from SCPs originating from the neural crest that migrate from the spinal cord to the intestine in this fish (El-Nachef and Bronner, 2020). Furthermore, SCPs can function as a source of mesenchymal cells that produce pulp cells and odontoblasts, as observed in a growing mouse tooth model (Kaukua et al., 2014). In addition to the above-mentioned cell types, neuroendocrine cells of the adrenal medulla (chromaffin cells) have also been shown to originate from the SCPs in mouse embryos and larvae of zebrafish, *D. rerio* (Furlan et al., 2017; Kamenev et al., 2021).

Moreover, it is known that chromaffin cells of the organ of Zuckerkandl and a portion of sympathetic neurons of the posterior paraganglia are largely derived from SCPs (Kastriti et al., 2019). A genetic tracing study has shown that SCPs are involved in the formation of glomus type I cells (primary oxygen-sensitive cells) of carotid bodies (Hockman et al., 2018). Also, a genetic tracing has recently shown that some SCPs are detached from nerve fibers and become mesenchymal cells that further differentiate into chondrocytes and mature osteocytes during the embryonic development in mice. Furthermore, in *D. rerio*, the chondrocyte development is also known to originate from SCPs, indicating that this process is evolutionarily conserved (Xie et al., 2019).

## Contribution of neural crest cells and Schwann cell precursors to gland development

**FIGURE 2:**
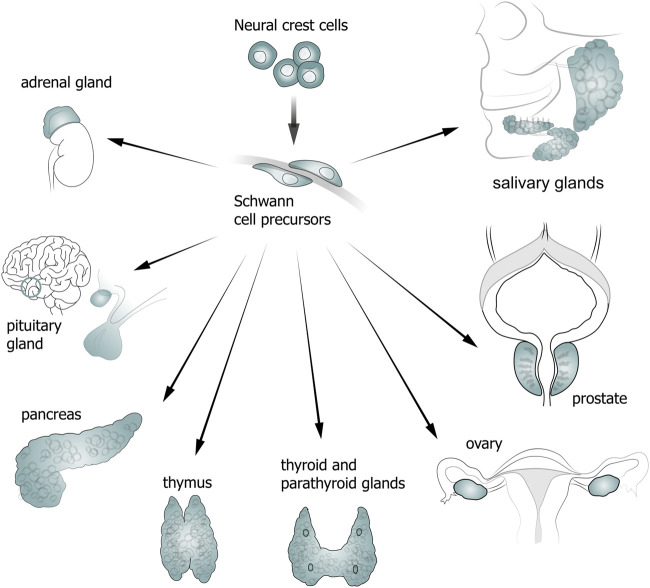
Diagram showing contribution of neural crest cells (NCCs) and Schwann cell precursors (SCPs) to gland development.

NCCs and SCPs play an important role in the development and functioning of various glands (Figure 2). Thus, a chimeric model of chicken/quail embryos has shown that most of the loose connective tissue of salivary glands originates from the neural crest (Nakamura, 1982). In addition, descendants of NCCs are found in the interlobular space and brain matter of the thymus, and also in the connective tissue of parathyroid glands (Suniara et al., 2000; Müller et al., 2008; Johansson et al., 2015).

## NCC descendants in thymus

The thymus has NC-derived mesenchyme which plays a crucial role in the growth and differentiation of glandular epithelium and T cells (Bockman and Kirby, 1984; Foster et al., 2008). In 12-day mouse embryo, NCCs migrated to the thymus region. Ablation of the neural crest in a region between the otic placode and the posterior part of somite 3 prevented the thymus formation or caused its underdevelopment. As the study showed, the number of cells positive to neural crest markers is reduced in thymus tissues, and the size of the forming thymus is directly related to the number of NCCs (Yamazaki et al., 2005).

Removal of perithymic mesenchyme from 12-day mouse embryo lobes blocked most lymphocytes at the CD4^–^CD8^–^ stage of development. However, if the thymus lobes were left intact, generation of all T-cell subtypes occurred in them *in vitro*. Suniara and co-authors showed that mesenchymal cells directly regulate lymphopoiesis and that a lack of the intrathymic network of NC-derived fibroblasts and their process of extracellular matrix formation in the thymus leads to a decrease in lymphoid tissue development. Furthermore, it is the extracellular matrix that is necessary for integrin and/or cytokine interactions during the thymus development (Suniara et al., 2000).

Yamazaki and co-authors reported that multipotent cells originating from the neural crest are found in the embryonic thymus at stages E14.5 and E15.5, but are absent from the thymus at stage E17.5. The authors concluded that the presence of NC-derived cells in the thymus is important for its early embryonic development (Yamazaki et al., 2005).

In 2008, Foster and co-authors used Wnt1^Cre^R26^eYfp^ and Sox10^Cre^R26^eYfp^ transgenic mice to show that NC-derived cells populate the thymus primordiumand remain in it during the embryonic development and until 9–10 months of age. In addition, the authors showed that NC-derived cells differentiate into perivascular cells involved in the formation of the blood–thymus barrier (Foster et al., 2008).

In the same year, Müller and co-authors also found NC-derived cells in the adult thymus. NC-derived mesenchyme, according to the authors, may take part in performing specific functions of the blood–thymus barrier in the adult thymus, and are also part of the microenvironment providing maintenance of migrating cells (Müller et al., 2008).

Myoid cells of the thymus are suggested to originate from the neural crest. This hypothesis was advanced by Nakamura and Ayer-Le Liére (1986) whose study, as well as the first studies by Nicole Le Douarin on the neural crest, was based on a hybrid model of chicken embryo, where part of NCCs were replaced by NCCs of quail at the same stage of development. The NC origin of myoid cells in birds was confirmed subsequently, in 2015 (Bódi et al., 2015).

## NCC descendants in thyroid and parathyroid glands

Early studies showed that calcitonin-producing thyroid cells originate from NCCs (Le Lievre and Le Douarin, 1970; Le Douarin and Le Lievre, 1970). This seminal study of neural crest cells, where the authors claimed that NCCs carry out invasion of ultimobranchial bodies prior to their fusion with the thyroid gland bud, was performed on chimeric avian embryos.

However, until 2007, there was no evidence whether the same process occurs in mammalian embryonic development. Kameda and co-authors showed that neither ectomesenchymal cells nor neural crest neuronal cells invade the ultimobranchial body primordium at stages E11.5–E12.5 (Kameda et al., 2007).

In 2015, Johansson and co-authors, using a genetic mouse model, confirmed and extended the previous idea that Wnt1^+^ NCCs contribute to the thyroid connective tissue development but are not a source of C cells in the mouse embryonic thyroid. Genetic tracing allowed the authors of this study to conclude that C cells are of endodermal origin (Johansson et al., 2015).

## NCC descendants in pituitary gland

The anterior part of the pituitary gland originates from the adenohypophyseal placode (Ueharu et al., 2017). It was long believed that NCCs differentiate into interstitial cells of the adenohypophysis. The presence of interstitial cells (from NCCs) in the developing anterior lobe of the pituitary gland was analyzed using chimeric chicken/quail embryos (Couly and Le Douarin, 1987). Ablation of NCCs using the Wnt1^Cre^ and P0^Cre^ mouse strains showed that these cells are involved in pituitary vascularization, thus, giving rise to pericytes (Davis et al., 2016). The contribution of NCCs to the cerebral vasculature was previously described from mice by several research teams using the P0-Cre and human tissue plasminogen activator (HtPA)-Cre reporter lines (Pietri et al., 2003; Nishiyama et al., 2012).

Ueharu et al. (2018), using genetic tracing in a mouse P0-Cre/EGFP model, showed that NCCs invade the adenohypophysis in several stages (waves). In a study by Kato with co-authors based on a S100β/GFP-TG rat model, NCCs populated the pituitary gland in several successive waves (Kato et al., 2021). The first wave was detected in mice at stage E9.5, when the pituitary primordium begins to form with adenohypophyseal placode cells; the second wave of invasion was observed at stage E14.5, when vasculogenesis proceeds from the Atwell’s recess. Genetic tracing of NCCs showed that they are capable of differentiating into both pericytes and all hormone-producing cell lineages of the adenohypophysis. According to the results of this study, NCCs contribute to pituitary organogenesis and vasculogenesis together with placode cell derivatives. In addition, the same team of researchers, using a different model, confirmed that SOX10-positive cells found in the pituitary gland are positive to another marker of NCCs, p75NTR. Immunohistochemical staining with antibodies to SOX10 during the pituitary development demonstrated the spatial and temporal pattern of localization of SOX10-positive cells in the posterior, intermediate, and anterior lobes (Ueharu et al., 2018).

## NCC descendants in pancreas

As Kirchgessner and co-authors showed on a model of rat intestine and pancreas explants, NCCs migrate to the intestine, and only after that continue their migration to the pancreas, where they give rise to intrapancreatic ganglion neurons (Kirchgessner et al., 1992). It was also reported that NCCs migrating into the pancreas differentiate into sympathetic, parasympathetic, and sensory neurons (Ahren, 2000).

Descendants of NCCs are known to be involved in pancreatic development. Smith (1975) showed that these cells are capable of differentiating into Schwann cells that surround the islets of Langerhans.

Signals from NCCs regulate the mass proliferation and maturation of pancreatic beta cells (Nekrep et al., 2008; Plank et al., 2011). Co-culturing of NCCs and pancreatic islets *in vitro* promoted the regeneration of functional beta cells (Olerud et al., 2009). NCC descendants migrating to the pancreatic region may be important for maintaining beta cell function, development, and maturation. Shimada et al. (2012) reported that NCC descendants maintain beta cell signaling at each stage of the pancreatic development. An earlier study showed that by 15.5 days post-coitum, neural crest-derived neurons were localized close to insulin-expressing clusters of cells and then contacted 98.9% of beta cell clusters on postnatal day 1 (Plank et al., 2011). However, the results obtained by the team of Shimada with co-authors showed that neural crest derivatives at late stages of embryonic development are closer to alpha cells than to beta cells in the pancreas. According to them, NCC derivatives surround the endocrine cells of the pancreas and influence them through juxtacrine or paracrine regulation (or both) (Shimada et al., 2012).

A number of studies carried out in the 1970s to elucidate the possible embryonic origin of pancreatic endocrine cells from NCCs were largely based on chimeric chicken/quail embryo models. Pictet et al. (1976), who investigated the origin of the islets of Langerhans, cultured rat embryos *in vitro* at early stages of development after removal of the neuroectoderm primordium. Interestingly, the pancreas developed under these conditions, and insulin-producing cells were observed. According to Andrew (1976) who used the chimeric model described above, neuroectodermal cells from the trunk never migrate to the pancreas. Andrew et al. (1986) and Fontaine and Le Douarin (1977), based on the same chimeric model, showed that NCCs migrating to the pancreas originated from the vagal region but did not differentiate into insulin-, glucagon-, somatostatin-, or avian pancreatic polypeptide- (APP) producing cells.

## NCC descendants in prostate

In 1999, Aumüller and co-authors found for the first time that human prostate neuroendocrine cells have a neurogenic origin, distinct from that of secretory and basal cells of the gland (Aumüller et al., 1999). Using a Wnt1-Cre/ROSA26-YFP mouse model and abortive human material, Szczyrba and co-authors showed that 64% of mouse prostate neuroendocrine cells originate from NCCs (Szczyrba et al., 2017).

## NCC descendants in sex glands

Single NCCs colonize the ovary at around E16.5 and differentiate into neurons and glia that give rise to the entire ovarian neural network. In contrast, NCCs do not infiltrate the testis. Theca cells, derived from the mesenchyme and steroidogenic cells of the ovary, also migrate into the ovary between E17.5 and P5 in a pattern very similar to that of the ovarian innervation development. In the ovary, nerve projections are located in close proximity to the theca cell layer of growing follicles, where they may be involved in stimulating theca or smooth muscle cells during the follicle growth and ovulation (McKey et al., 2019).

## NCC descendants in adrenal glands and organ of Zuckerkandl

Chromaffin cells of the adrenal medulla originate from Schwann cell precursors (SCPs) that are descendants of NCCs (Furlan et al., 2017).

SCPs migrate along axons of cholinergic preganglionic neurons in the spinal cord, moving towards the developing sympathoadrenal primordium (Lumb et al., 2018). Upon reaching the rudiment (stages E11.5 and E12.5 in mouse), the SCPs begin to differentiate into chromaffin cells of the adrenal medulla, passing through a transition state referred to as the “bridge”. It is worth mentioning that most SCPs do not transdifferentiate subsequently but continue the transformation into satellite glial cells located next to neuronal bodies in peripheral ganglia or into Schwann cells covering peripheral nerves. A little later in development, some of the immature, nerve-associated Schwann cells begin to myelinate, while the rest remain dormant on nerves as Remak cells or non-myelinating Schwann cells (Jessen and Mirsky, 2005; Jessen et al., 2015).

A study based on transgenic mice showed that organ of Zuckerkandl cells can also originate from nerve-associated SCPs, similarly to adrenal chromaffin cells (Furlan et al., 2017).

## NCC descendants in submandibular salivary glands

Cells originating from NCCs were found in the submandibular salivary glands of adult mice. These cells form a kind of “islets” inside the glands and express various markers characteristic of NCCs such as Sox10 and Ednrb. Furthermore, as was shown on a Wnt1-Cre/R26R lacZ mouse model, the mesenchyme of embryonic submandibular salivary glands originates entirely from the cranial region of the neural crest (Jaskoll et al., 2001; Takahashi et al., 2014).

## Conclusion

Neural crest cells (NCCs) are often referred to as the “fourth germinal layer” because of their broad potential to differentiate into a variety of adult cells, ranging from mesenchymal cells to various types of neurons. The emergence of NCCs in the course of evolution has contributed to the generation of a large number of new structures or to the increased complexity of existing ones in chordate organisms. NCCs take an active part in the development of various systems of the body, including the formation of endocrine glands, regulating their proper embryonic development and giving rise to neuroendocrine cells, neurons, and various types of glial cells. Schwann cell precursors, being also multipotent descendants of NCCs, are involved in the gland formation, but many questions as to their role in endocrine embryogenesis remain unaddressed. Despite the ongoing active research in the field of genome evolution and the development of the New Head hypothesis, the origin and evolution of the neural crest and its descendants remains not entirely clear.

Understanding the cellular mechanisms of various organs formation during embryonic development is extremely important for compiling a complete picture of normal embryogenesis, as well as for identifying targets in the treatment of diseases of the adult body and searching for a cellular source for replacement therapy. NCCs and SCPs are among the key participants in the formation of various parts of the nervous and endocrine systems, which makes them an important object for further study.
